# Quality of life and treatment satisfaction with pharmacological interventions in Chinese adults with chronic pain due to osteoarthritis

**DOI:** 10.1186/s12891-021-04012-2

**Published:** 2021-02-13

**Authors:** Qingyun Xue, Huibin Long, Jianhao Lin, Dongping Du, Jin Zhou, Jinwei Chen, Shu li, Yanlei Zhang, Yan Cheng, Xiao Ma, Zhiyi Zhang

**Affiliations:** 1 Department of Orthopedics, Beijing Hospital, Beijing, China; 2 Arthritis Clinic and Research Center, Peking University People’s Hospital, Beijing, China; 3 Department of Pain Management, Shanghai Jiao Tong University Affiliated Sixth People’s Hospital, Shanghai, China; 4 Department of Rheumatology and Immunology, The Second Xiangya Hospital of Central South University, Changsha, China; 5 Eli Lilly and Company, Shanghai, China; 6 Department of Rheumatology and Immunology, The First Affiliated Hospital of Harbin Medical University, Harbin, China

**Keywords:** Knee osteoarthritis, Chronic pain, Health-related quality of life, Treatment satisfaction, Pain severity

## Abstract

**Background:**

Aim of this multicenter, observational, cross-sectional study was to evaluate health-related quality of life (HRQoL) and treatment satisfaction of current medications in Chinese knee OA patients.

**Methods:**

Brief Pain Inventory (BPI), Treatment Satisfaction Questionnaire (TSQM-1.4), and HRQoL (EQ-5D-5L) were assessed in total of 601 OA of knee patients. Impact on QoL (EQ-5D-5L) and treatment satisfaction (TSQM-1.4) by BPI-Severity score (< 4 and ≥ 4) were presented using mean standard deviations (SDs) and were compared using a t-test. For each of self-assessed health EQ-5D-5L and TSQM, a linear regression model was used to estimate the regression coefficient along with corresponding 95% confidence interval (CI) for BPI-Severity.

**Results:**

Mean score of EQ-5D-5L of patients with BPI-Severity ≥4 was significantly lower than those with BPI-Severity < 4. All the scores of TSQM in 4 dimensions were lower in patients with BPI-Severity ≥4 than in those with BPI-Severity < 4. Both HRQoL scores and TSQM scores showed a statistically significant decreasing trend with increasing BPI-Severity pain score.

**Conclusion:**

Chronic knee OA pain has a significant impact on patients’ HRQoL. More severe patients with OA were less satisfied with current treatments.

**Supplementary Information:**

The online version contains supplementary material available at 10.1186/s12891-021-04012-2.

## Background

Osteoarthritis (OA) is one of the most prevalent chronic musculoskeletal disorders and a leading cause of disability worldwide, especially among the elderly [1]. Globally, the prevalence of knee OA in men is lower compared to women, with 9.6% of men and 18% of women aged over 60 years affected [1, 2]. In China, the prevalence of radiographic OA was 42.8% in women and 21.5% in men; whereas, symptomatic OA occurred in 15% of women and 5.6% of men. The prevalence of radiographic and symptomatic OA in Chinese men was similar to that in white men in the United States (US). However, Chinese women had a higher prevalence of radiographic and symptomatic OA than women in the US [2, 3].

Chronic pain is one of the most common health issues that exerts a significant social and financial burden on the individual and society. Patients with inadequate pain relief are more likely to have worse quality of life (QoL), greater function loss, and greater pain interference with daily activities [4]. OA is a leading cause of deteriorated QoL due to chronic pain [5, 6]. Compared with the radiographic OA without pain, painful OA has been associated with higher cardiovascular risk and mortality [7]. Pain is recognized as one of the hallmark symptoms in OA and is a common reason patients seek medical attention. Mechanisms underlying chronic pain include a complex interaction of physiological, emotional, cognitive, social, and environmental factors [8]. When considering the complex nature of chronic pain, treatment often necessitates the use of a blend of different approaches. In terms of nonsurgical standard interventions for OA, multimodal pain management is a comprehensive treatment of complex chronic pain syndromes that includes 4 core disciplines of multimodal pain management: pain medicine, psychotherapy, exercise therapy (including physiotherapy), and assistant medical professions including nurses. Multimodal pain management protocols aim to address pain control, facilitate functional recovery, and maintain patient satisfaction [9, 10]. According to guidelines for diagnosis and treatment of OA in China, the purpose of OA treatment is to relieve pain; slow disease progression; deformity correction; improve or restore joint function; and improve patients’ QoL. The guidelines recommend a stepwise approach for management of OA which include, a) basic treatment such as patient education (increase disease awareness, avoid bad life/work habit such as long-time running or jumping, avoid climbing stairs or mountains, lose weight), exercise therapy (low-intensity acrobatic exercise; muscle strengthening training; joint function training), physical therapy (heat, therapeutic cooling, acupuncture, massage) and motion assistance (cane, joint brace); b) medications (NSAIDs, glucocorticoid, sodium hyaluronate, symptomatic slow-acting drugs for OA); and c) surgery [11].

Patient-reported outcome is an important consideration in the treatment of patients with OA. All aspects of QoL are compromised when pain is inadequately treated, and effective pain relief has been shown to improve health-related quality of life (HRQoL) [12, 13]. When patients with OA were asked to rank aspects of QoL impacted by their condition, they highlighted enjoyment of life, emotional well-being, fatigue, weakness, and sleep-related problems as the most important areas they would consider when evaluating the success of their pain treatment [14]. The pain caused by OA can have a substantial impact on patients’ QoL [11–13]. In a 2012 online survey of patients with OA in the United Kingdom [15], 52% of the 2001 respondents reported that OA had a large impact on their life, 71% reported having persistent pain even after taking their prescribed pain medication, and 12% said their pain was often unbearable. In a cross-sectional study conducted in 2014 by Kantar Health, only 14% of patients in Japan with diagnosed pain who suffered from joint pain were highly satisfied with their pain medications [4]. Furthermore, a multinational longitudinal survey showed that patients with inadequate pain relief were more likely to have a worse QoL, greater function loss, and greater pain interference [4].

Patient satisfaction is an important indicator of the quality of care provided to patients with OA [16]. Patient-reported outcomes, such as HRQoL and patient satisfaction, were used to capture patients’ experience of chronic disease and can support the physician in clinical practice to facilitate patient-centered care [17]. Thus, QoL and treatment satisfaction assessments are crucial to evaluating the clinical effectiveness of treatment in OA.

Little is known about the impact of chronic knee OA pain on HRQoL and treatment satisfaction in a real-world setting in China. Therefore, the cross-sectional survey presented in this article has been designed to understand the impact of chronic knee OA pain on HRQoL and to evaluate treatment satisfaction of current medications among Chinese patients with knee OA.

## Methods

### Study design and subjects

This site-based, multicenter, observational, cross-sectional study in China enrolled 601 outpatients with knee OA from 2 orthopedics, 2 rheumatology, and 1 pain department in 5 tertiary hospitals from March to October 2018. Written informed consent was obtained from each patient before they participated in any study-related procedures.

Chinese adult patients (aged ≥40 years) with diagnosed knee OA experiencing chronic pain for at least 3 months and receiving oral medications during the past 12 months were eligible for the study. Patients with rheumatoid arthritis or other inflammatory arthritis; knee pain caused by other diseases (eg, traumatic fracture history or tumor); mental illness, including cognitive disorders such as Alzheimer’s disease, schizophrenia; and bedridden patients who were undergoing knee replacement surgery were excluded. Patients with pain level higher than knee pain due to cancer or other reasons such, as gout and chondrocalcinosis, were also excluded. Socio-demographics, disease characteristics, Brief Pain Inventory (BPI), treatment information, and patient responses to HRQoL (5-level of Chinese Quality of Life-5 Dimensions version [EQ-5D-5L] and self-assessed health) and Treatment Satisfaction Questionnaire for Medication (TSQM-1.4) interviews were also assessed.

### Measures

#### Patient characteristics

The characteristics measured were age, sex, body mass index, ethnicity, employment status, education status, insurance status, and comorbidity (detailed patient comorbidities are presented in Table 1). The following OA characteristics were measured for each enrolled patient: age and location at first diagnosis, current department of visits, number and location of painful sites, and severity of pain. The average number of weekly days of paid work or housework lost due to OA was also recorded. In addition, information related to the current treatment for OA pain management (including non-pharmacotherapy) was collected from each enrolled patient.

**Table 1:** Patient demographics and baseline characteristics

Characteristics	***N*** = 601
**Age, mean (SD)**	61.77 (9.53)
**Body mass index, mean (SD)**	24.66 (3.16)
**Gender,** ***n*** **(%)**	
Male	149 (24.79)
Female	452 (75.21)
**Nationalities,** ***n*** **(%)**	
Han	587 (97.67)
Others	14 (2.33)
**Working status,** ***n*** **(%)**	
Unemployed	37 (6.17)
Part-time	7 (1.17)
Full-time	144 (24)
Retired	412 (68.67)
**Educational status,** ***n*** **(%)**	
Below senior high school	293 (48.75)
Senior high school	137 (22.8)
Junior college	88 (14.64)
Undergraduate	76 (12.65)
Postgraduate or above	7 (1.16)
**Insurance types,** ***n*** **(%)**	
Urban resident basic medical insurance	177 (29.45)
Urban employee basic medical insurance	242 (40.27)
New rural cooperative medical system	158 (26.29)
Commercial health insurance	5 (0.83)
Uninsured	19 (3.16)
**Comorbidity,** ***n*** **(%)**	
Any comorbidities	331 (55.07)
Hypertension	239 (39.77)
Coronary heart disease	75 (12.48)
Myocardial infarction	2 (0.33)
Stroke	12 (2)
Cerebral hemorrhage	1 (0.17)
Gastritis	86 (14.31)
Nephropathy	15 (2.5)
Diabetes	92 (15.31)
Stomach or duodenal ulcers	20 (3.33)

*n* number of subjects, *SD* standard deviation

#### Outcome measures

The BPI is a validated self-reported questionnaire that assesses pain severity using the Numerical Rating Scale for Pain Intensity (NRS-PI, 0 to 10 scale, where 0 = no pain and 10 = worst possible pain) for the conditions of worst, least, and average pain, as well as “pain right now”. The 5-level Chinese Quality of Life-5 Dimensions version (EQ-5D-5L) [18] comprises 5 dimensions: mobility, self-care, usual activities, pain/discomfort, and anxiety/depression. Each dimension has 5 levels: no problems, slight problems, moderate problems, severe problems, and extreme problems. Self-health care assessment was performed using the EuroQol (EQ) visual analogue scale (EQ VAS). The EQ VAS self-rating records the respondent’s own assessment of their health status on a 20-cm vertical VAS with endpoints labelled ‘the best health you can imagine’ and ‘the worst health you can imagine.’ [19] The TSQM was designed to assess treatment satisfaction for patients with chronic diseases. The TSQM 1.4 is a 14-item psychometrically robust and validated instrument consisting of 4 scales: effectiveness, side effects, convenience, and global satisfaction, each on a scale of 0–100 with higher scores indicating a higher level of satisfaction.

#### Statistical analyses

Demographic and clinical characteristics were assessed using frequencies and percentages for categorical variables and mean values and SDs for continuous variables (descriptive analysis) in the whole patient population. Impact on QoL (EQ-5D-5L) and treatment satisfaction (TSQM-1.4) by BPI-Severity score (< 4 and ≥ 4) were presented using mean (SD) and were compared using a t-test. For each of self-assessed health, EQ-5D-5L, and TSQM, a linear regression model was used to estimate the regression coefficient along with corresponding 95% confidence interval (CI) for BPI-Severity, adjusting for age (continuous), sex, body mass index (BMI), number of pain sites (continuous), and comorbidity (yes or no). We assessed the effect modification of comorbidity on a multiplicative scale by including interaction term between BPI-Severity and comorbidity in linear regression models. Additionally, we conducted the same analysis for the association between BPI-Pain interference and self-assessed health, EQ-5D-5L, and TSQM. Missing data were not analyzed. Statistical analyses were conducted using SAS 9.4 (SAS Institute, Cary, NC), and a 2-sided *P* value of 0.05 was considered statistically significant.

## Results

**Fig. 1:**
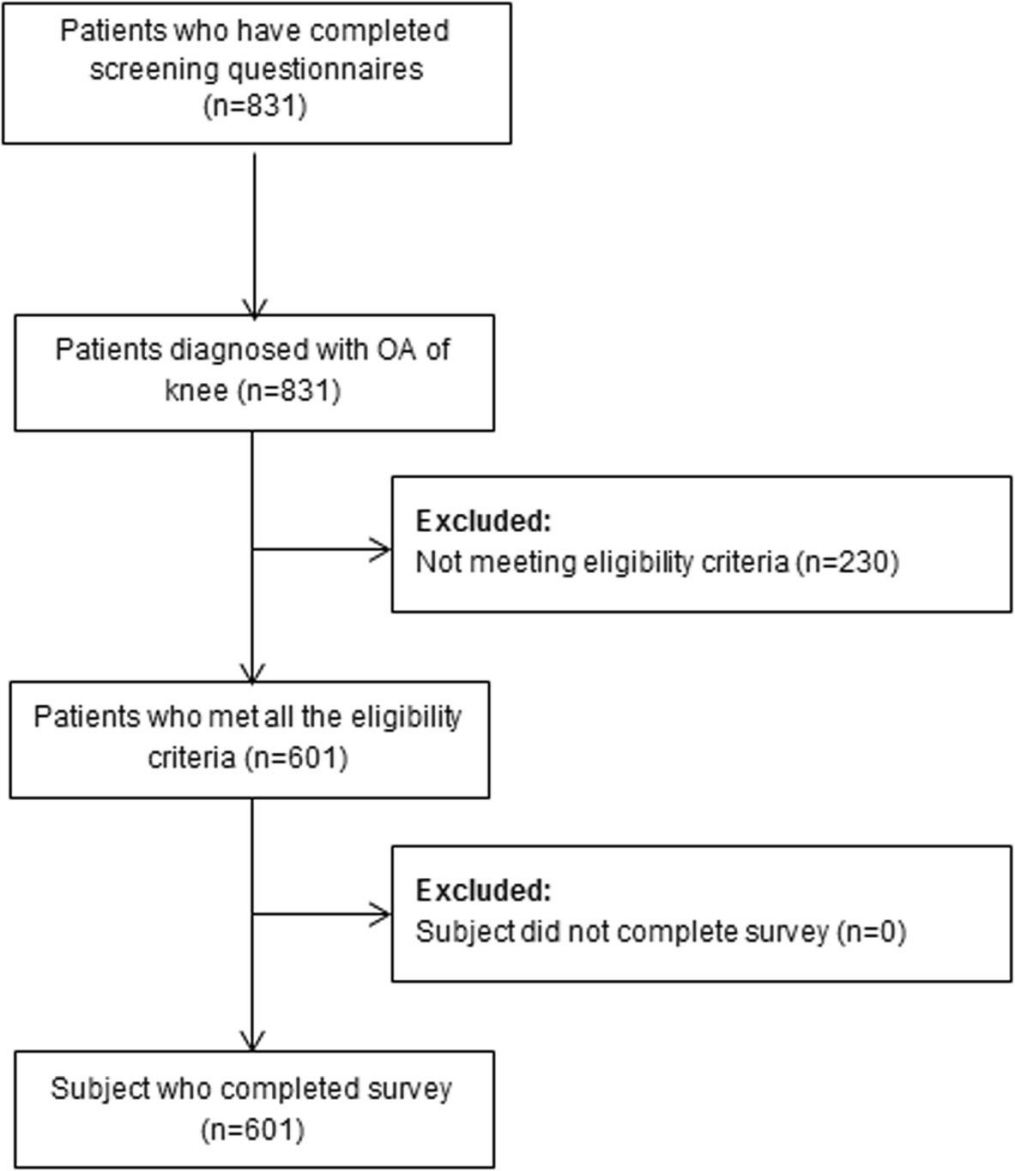
Flow chart of survey sampling. n: number of subjects; OA: osteoarthritis

A total of 601 patients met the eligibility criteria and completed this survey (Fig. 1). The mean (SD) age of enrolled patients was 61.77 (9.53) years and the majority of patients were female. More than 50% of patients had at least 1 comorbidity of gastrointestinal or cardiovascular disease (Table 1). The most commonly used current treatments for knee OA were oral medication, a patch or ointment, or intra-articular hyaluronic acid injection (Table 2). More than half of patients were rated with BPI-Severity ≥4. Pain interfered with work productivity, with 37.1% of patients self-reporting that more than 4 days/week of work or housework were lost due to OA pain.

**Table 2:** Clinical characteristics of knee osteoarthritis

Characteristics	***N*** = 601
**Age at first diagnosis, mean (SD)**	58.13 (9.62)
**Location at first diagnosis,** ***n*** **(%)**	
Unilateral knee	290 (48.33)
Bilateral knee	278 (46.33)
Others (shoulders, elbows, hips, etc.)	32 (5.33)
**Current department,** ***n*** **(%)**	
Rheumatology	155 (25.79)
Orthopedics	326 (54.24)
Pain	120 (19.97)
**Brief Pain Inventory score, mean (SD)**	
Pain Severity (full score: 10)	3.78 (1.62)
Pain Interference (full score: 10)	2.97 (1.70)
**Treatment pattern,** ***n*** **(%)**	
Oral medication (Western/traditional Chinese medicine)	469 (78.04)
Patch/ointment	271 (45.09)
Intra-articular hyaluronic acid injection	189 (31.45)
Intra-articular steroid injection	125 (20.8)
Physiotherapy (electrotherapy/hyperthermia)	88 (14.64)
Kinesitherapy (rehabilitation treatment)	22 (3.66)
Orthoses (cane, etc.)	4 (0.67)
Others	30 (4.99)
**Average weekly days of paid work or housework loss due to osteoarthritis, mean (SD) in the past month**	
0 day	235 (39.30)
1 day	44 (7.36)
2–3 days	97 (16.22)
≥ 4 days	222 (37.12)

*n* number of subjects, *SD* standard deviation

The mean score of EQ-5D-5L of patients with BPI-Severity ≥4 was significantly lower than those with BPI-Severity < 4 (0.62 vs 0.84, *P < 0.0001*) (Table 3). A similar trend was observed for self-assessed health outcomes, where the mean self-assessed health score of patients with BPI-Severity ≥4 was significantly lower than those with BPI-Severity < 4 (66.88 vs 73.8, *P < 0.0001*). There were statistically significant differences in all 4 domains of TSQM-1.4 between both patient subgroups (BPI-Severity ≥4 and < 4) (Table 4). The mean score of TSQM for the patients with BPI-Severity ≥4 was significantly lower than those with BPI-Severity < 4 for effectiveness (51.0 vs 57.8, *P < 0.0001*), side Effects (94.9 vs 97.2, *P = 0.0099*), convenience (60.2 vs 64.7, *P < 0.0001*), and global Satisfaction (57.7 vs 60.4, *P = 0.0402*). As shown by the TSQM score, treatment satisfaction was significantly lower in patients with BPI-Severity ≥4 than in those with BPI-Severity < 4.

**Table 3:** Impact on quality of life assessed using EQ-5D-5L questionnaire, Self-assessed health by BPI-Severity score (< 4 and ≥ 4)

Characteristics	All Patients Mean (SD)	Pain Severity < 4 (***n*** = 283) Mean (SD)	Pain Severity ≥ 4 (***n*** = 318) Mean (SD)	***P*** value
**Quality of life (EQ-5D-5L),** (full score: 1.00)	0.68 (0.23)	0.84 (0.13)	0.62 (0.22)	< 0.0001
**Self-assessed health (EQ VAS),** (full score:100)	70.62 (17.48)	73.8 (12.38)	66.88 (16.72)	< 0.0001

*BPI* Brief Pain Inventory, *EQ-5D-5L* EQ-5 dimension 5-level, *EQ VAS* EQ visual analogue scale, *n* number of subjects, *SD* standard deviation, *VAS* visual analog scale

**Table 4:** Treatment satisfaction assessed using TSQM-1.4 questionnaire by BPI-Severity score (< 4 and ≥ 4)

Characteristics	All Patients Mean (SD)	Pain Severity < 4 (***n*** = 283) Mean (SD)	Pain Severity ≥ 4 (***n*** = 318) Mean (SD)	***P*** value
TSQM-Effectiveness	54.2 (14.1)	57.8 (12.4)	51.0 (14.8)	< 0.0001
TSQM-Side Effects	96 (10.9)	97.2 (9.0)	94.9 (12.3)	0.0099
TSQM-Convenience	62.3 (10.4)	64.7 (10.8)	60.2 (9.6)	< 0.0001
TSQM-Global Satisfaction	59.0 (15.4)	60.4 (11.9)	57.7 (18.0)	0.0402

*BPI* Brief Pain Inventory, *n* number of subjects, *SD* standard deviation, *TSQM* Treatment Satisfaction Questionnaire for Medication (full score: 100)

The BPI-Pain Severity scores were inversely associated with the self-assessed health, EQ-5D-5L, and TSQM scores. In linear regression models adjusted for age, sex, BMI, number of pain sites, and comorbidity, HRQoL scores (self-assessed health [− 3.05; *P < 0.0001*] and EQ-5D-5L [− 0.08; *P < 0.0001*]) showed a significant decreasing trend with each unit increase in BPI-Severity pain score, indicating that reduction in knee pain was statistically significantly associated with improvements in HRQoL scores (Table 5). The score of TSQM also showed a significant decreasing trend in effectiveness: (− 2.75, 95%CI: − 3.46, − 2.04), side effects (− 0.65, 95%CI: − 1.22, − 0.08), convenience (− 1.31, 95%CI: − 1.84, − 0.77), and global satisfaction (− 1.25, 95%CI: − 2.05, − 0.45) with each unit increase in BPI-Severity pain score (Table 6), indicating that lower knee pain was significantly associated with higher TSQM effectiveness, side effects, convenience, and global satisfaction scores. Furthermore, the BPI-Pain severity scores in patients with and without comorbidity were also inversely associated with the self-assessed health, EQ-5D-5L, and TSQM scores. In linear regression models adjusted for age, sex, BMI, number of pain sites, and comorbidity, HRQoL scores in patients with and without comorbidity (self-assessed health [− 2.48 and − 3.84 in patients with and without comorbidity, respectively, *P*_*interaction*_ *= 0.0621*] and EQ-5D-5L [− 0.08 for both in patients with and without comorbidity, *P*_*interaction*_ *= 0.5883*]), indicated that comorbidity does not modify the association between BPI-Pain and HRQoL scores (Table S1). Similarly, the score of TSQM also showed a decreasing trend per BPI-Severity score (effectiveness: − 2.66 and − 2.83, *P*_*interaction*_ *= 0.9557*; side effects: − 0.19 and − 1.20, *P*_*interaction*_ *= 0.0715*; convenience: − 1.56 and − 1.01, *P*_*interaction*_ *= 0.4260*; and global satisfaction: − 1.22 and − 1.22, *P*_*interaction*_ *= 0.8612* in patients with and without comorbidity, respectively), indicating that comorbidity does not modify the association between BPI-Pain and TSQM scores, (Table S2).

**Table 5:** The association between BPI and HRQoL

EQ-5D-5L and self-assessed health per BPI	Parameter estimate^**a**^	95% CI	***P*** value	Parameter estimate^**b**^	95% CI	***P*** value
EQ-5D-5L	−0.08	(−0.09, − 0.08)	< 0.0001	− 0.08	(− 0.09, − 0.07)	< 0.0001
Self-assessed health	−3.31	(−4.03, −2.59)	< 0.0001	− 3.05	(− 3.78, − 2.32)	< 0.0001

^a^Adjusted for age. ^b^Adjusted for age, sex, BMI, number of pain sites, and comorbidity. *BMI* body mass index, *BPI* Brief Pain Inventory, *CI* confidence interval, *EQ-5D-5L* EQ 5 dimension-5-level, *HRQoL* health-related quality of life

**Table 6:** The association between BPI and TSQM

TSQM per BPI	Parameter estimate^**a**^	95% CI	***P*** value	Parameter estimate^**b**^	95% CI	***P*** value
TSQM- Effectiveness	−2.64	(− 3.33, − 1.94)	< 0.0001	− 2.75	(− 3.46, − 2.04)	< 0.0001
TSQM- Side effect	−0.59	(− 1.14, − 0.03)	0.0381	−0.65	(− 1.22, − 0.08)	0.0254
TSQM-Convenience	−1.42	(− 1.94, − 0.90)	< 0.0001	−1.31	(− 1.84, − 0.77)	< 0.0001
TSQM- Global satisfaction	−1.08	(− 1.86, − 0.29)	0.0073	−1.25	(− 2.05, − 0.45)	0.0022

^a^Adjusted for age. ^b^Adjusted for age, sex, BMI, number of pain sites, and comorbidity. *BMI* body mass index, *BPI* Brief Pain Inventory, *CI* confidence interval, *TSQM* Treatment Satisfaction Questionnaire for Medication

In addition, we also conducted an analysis for BPI-Pain interference. Mean scores for self-assessed health, EQ-5D-5L and TSQM (4 dimensions) in patients with BPI-Interference ≥3 were lower than those with BPI-Interference < 3 (Table S3 and Table S4). Both HRQoL scores and TSQM scores showed a statistically significant decreasing trend with increasing BPI-Interference pain score (Table S5 and Table S6). Similarly, in patients with and without comorbidity HRQoL scores and TSQM scores showed a decreasing trend with BPI-Interference, indicating comorbidity does not modify BPI-Interference and HRQoL or TSQM scores. (Table S7 and Table S8).

## Discussion

The cross-sectional survey presented in this article is the first large-scale, multicenter real-world study to explore the impact of OA pain on HRQoL and treatment satisfaction among Chinese patients with OA]. The results of this study, show that chronic pain has not been well managed since 78% of Chinese patients with OA who were treated with pharmacological therapy combined with other therapies still experienced moderate-to-severe pain (BPI ≥ 4) and significantly lower HRQoL and treatment satisfaction. Moreover, more than 35% of patients self-reported that they lost more than 4 days/week of work due to OA pain. These observations indicate that the patients with OA were not satisfied with current treatments. The cross-sectional survey results suggest that patients with moderate-to-severe OA pain had significantly lower HRQoL and treatment satisfaction scores as compared to patients with mild OA pain. Overall, pain severity plays an important role in predicting HRQoL and treatment satisfaction in Chinese patients with knee OA. Also, the study results suggest that increased pain severity is associated with a decrease in the levels of HRQoL and treatment satisfaction among Chinese patients with OA. Reduction in knee pain was statistically significantly associated with improvements in HRQoL and treatment satisfaction among Chinese patients with OA.

The analysis results suggest that pain severity plays an important role in predicting HRQoL, and our findings are consistent with the previous studies [20–22]. A published study demonstrated that patients experiencing OA pain in both knees have poorer HRQoL compared to patients with unilateral knee pain or no knee pain [23]. A population-based study in Japan revealed that patients with severe knee OA had significantly lower physical HRQoL than those with mild and moderate knee OA [24]. A large population-based cohort study from southern Sweden also confirmed that participants with knee OA (defined either clinically or radiographically) reported lower HRQoL scores than those with no knee OA [25]. The results of another study showed that patients with radiographic knee OA had considerably lower scores in all subgroups of SF-36 compared with healthy controls [26]. The results obtained from a cross-sectional study revealed that the lower HRQoL scores were associated with increased pain severity in patients with knee OA [27].

Patient satisfaction with treatment is essential in OA and is a measure of therapeutic effectiveness [10, 28]. In this study, TSQM scores in 4 dimensions were significantly lower in patients with OA with moderate-to-severe pain intensity (BPI-Severity ≥4) than in those with mild OA pain intensity (BPI-Severity < 4). This indicates that treatment satisfaction was found to be higher in OA patients with lower pain, which is consistent with an earlier study showing that decreased pain was associated with increased treatment efficacy and, thereby, patient satisfaction [28]. Thus, switching treatments to achieve lower pain levels might enhance treatment satisfaction among patients with knee OA. Stahmer et al. [29] reported that patient satisfaction with pain management is associated with the amount of pain relief achieved. Moreover, the findings regarding pain as an important factor in predicting treatment satisfaction may be extrapolated to patients with knee OA globally. In summary, pain severity has a great impact on HRQoL and treatment satisfaction in Chinese patients with knee OA. Pain relief may help improve patients’ HRQoL and treatment satisfaction. This real-world study provided the evidence that relieving pain should be the first choice of therapy for knee OA.

Our study does have some limitations. Since data were derived from a cross-sectional survey, the association between knee OA pain severity and HRQoL and treatment satisfaction cannot be viewed as causal. Longitudinal studies are needed to examine the relationship between knee OA pain severity and HRQoL and treatment satisfaction. For the loss of workdays due to OA pain, we could not retrieve specific data regarding the types of work involved, hence, the results need to be interpreted with caution. Moreover, the study was conducted at 5 tertiary hospitals in China, and no randomization mechanism was used in their selection; hence, it is difficult to generalize the findings.

## Conclusions

Chronic pain due to OA, especially in those patients with moderate-to-severe pain, has a significant impact on patients’ HRQoL. In our study, patients with more severe OA were less satisfied with current treatments. Appropriate pain management in China is important in improving HRQoL and the treatment satisfaction for medication.

## Supplementary Information

**Additional file 1: Table S1.** The association between BPI-Severity score and HRQoL in patients with/without comorbidity. **Table S2.** The association between BPI-Severity score and TSQM in patients with/without comorbidity. **Table S3.** Impact on quality of life assessed using EQ-5D-5L questionnaire and self-assessed health by BPI-Interference score (< 3 and ≥ 3). **Table S4.** Treatment satisfaction assessed using TSQM-1.4 questionnaire by BPI-Interference score (< 3 and ≥ 3). **Table S5.** The association between BPI-Interference score and HRQoL. **Table S6.** The association between BPI-Interference score and TSQM. **Table S7.** The association between BPI-Interference score and HRQoL in patients with/without comorbidity. **Table S8.** The association between BPI-Interference score and TSQM in patients with/without comorbidity

## Data Availability

The datasets generated and/or analyzed during the current study are available from the author Xiao Ma (ma_xiao4@lilly.com) on reasonable request.

## Abbreviations

BPI: Brief Pain Inventory
CI: Confidence interval
EQ VAS: EuroQol visual analogue scale
EQ-5D-5L: The 5-level Chinese Quality of Life-5 Dimensions version.
HRQoL: Health-related quality of life
NRS-PI: Numerical Rating Scale for Pain Intensity
OA: Osteoarthritis
QoL: Quality of life
SDs: Standard deviations
TSQM: Treatment Satisfaction Questionnaire
US: United States
