# Supplementary material for: Quality of life and treatment satisfaction with pharmacological interventions in Chinese adults with chronic pain due to osteoarthritis
Source: BMC Musculoskelet Disord. 2021 Feb 13;22:178. doi: 10.1186/s12891-021-04012-2 (PMC7882067; doi:10.1186/s12891-021-04012-2)
Supplement: Supplementary file 1 — Additional file 1: Table S1. The association between BPI-Severity score and HRQoL in patients with/without comorbidity. Table S2. The association between BPI-Severity score and TSQM in patients with/without comorbidity. Table S3. Impact on quality of life assessed using EQ-5D-5L questionnaire and self-assessed health by BPI-Interference score (< 3 and ≥ 3). Table S4. Treatment satisfaction assessed using TSQM-1.4 questionnaire by BPI-Interference score (< 3 and ≥ 3). Table S5. The association between BPI-Interference score and HRQoL. Table S6. The association between BPI-Interference score and TSQM. Table S7. The association between BPI-Interference score and HRQoL in patients with/without comorbidity. Table S8. The association between BPI-Interference score and TSQM in patients with/without comorbidity [file 12891_2021_4012_MOESM1_ESM.docx]

**Supplementary appendix**

| **EQ-5D-5L and Self-assessed health per BPI-Severity score** | **With comorbidity** | | | |  | **Without comorbidity** | | | | | **P value^c^** |
| --- | --- | --- | --- | --- | --- | --- | --- | --- | --- | --- | --- |
|  | **Parameter estimate^a^** | **95% CI** | **Parameter estimate^b^** | **95% CI** |  | **Parameter estimate^a^** | **95% CI** | **Parameter estimate^b^** | | **95% CI** |  |
| EQ-5D-5L | -0.08 | (-0.1, -0.07) | -0.08 | (-0.09, 0.07) |  | -0.08 | (-0.09, -0.07) | | -0.08 | (-0.09, -0.07) | 0.5883 |
| Self-assessed health | -2.76 | (-3.74, -1.79) | -2.48 | (-3.45, -1.51) |  | -3.99 | (-5.08, -2.9) | | -3.84 | (-4.97, -2.72) | 0.0621 |

**Table S1. The association between BPI-Severity score and HRQoL in patients with/without comorbidity**

aAdjusted for age. bAdjusted for age, sex, BMI, and number of pain sites. cAdjusted for age, sex, BMI, number of pain sites, and comorbidity. BMI: body mass index; BPI: Brief Pain Inventory; CI: confidence interval; EQ-5D-5L: EQ 5 dimension-5-level; HRQoL: health-related quality of life.

| **TSQM per BPI-Severity score** | **With comorbidity** | | | |  | **Without comorbidity** | | | | | **P value^c^** |
| --- | --- | --- | --- | --- | --- | --- | --- | --- | --- | --- | --- |
|  | **Parameter estimate^a^** | **95% CI** | **Parameter estimate^b^** | **95% CI** |  | **Parameter estimate^a^** | | **95% CI** | **Parameter estimate^b^** | **95% CI** |  |
| TSQM-effectiveness | -2.56 | (-3.49, -1.63) | -2.66 | (-3.63, -1.7) |  | -2.70 | (-3.76, -1.64) | | -2.83 | (-3.92, -1.75) | 0.9557 |
| TSQM-side effect | -0.08 | (-0.83, 0.68) | -0.19 | (-0.97, 0.6) |  | -1.17 | (-1.99, -0.35) | | -1.20 | (-2.04, -0.35) | 0.0715 |
| TSQM-convenience | -1.66 | (-2.39, -0.94) | -1.56 | (-2.31, -0.81) |  | -1.14 | (-1.89, -0.39) | | -1.01 | (-1.78, -0.25) | 0.4260 |
| TSQM-global satisfaction | -0.90 | (-1.96, 0.15) | -1.22 | (-2.3, -0.15) |  | -1.05 | (-2.25, 0.14) | | -1.22 | (-2.44, 0) | 0.8612 |

# Table S2. The association between BPI-Severity score and TSQM in patients with/without comorbidity

aAdjusted for age. bAdjusted for age, sex, BMI, and number of pain sites. cAdjusted for age, sex, BMI, number of pain site, and comorbidity. BMI: body mass index; BPI: Brief Pain Inventory; CI: confidence interval; TSQM: Treatment Satisfaction Questionnaire for Medication

**Table S3. Impact on quality of life assessed using EQ-5D-5L questionnaire and self-assessed health by**

**BPI-Interference score (<3 and ≥3)**

| **Characteristics** | **All Patients Mean (SD)** | **Pain Interference <3 (n=339) Mean (SD)** | **Pain Interference ≥3 (n=262) Mean (SD)** | **P value** |
| --- | --- | --- | --- | --- |
| Quality of life (EQ-5D-5L), (full score: 1.00) | 0.68 (0.23) | 0.84 (0.10) | 0.56 (0.22) | <0.0001 |
| Self-assessed health (EQ VAS), (full score:100) | 70.62 (17.48) | 74.63 (12.31) | 64.32 (16.61) | <0.0001 |

BPI: Brief Pain Inventory; EQ-5D-5L: EQ-5 dimension 5-level; EQ VAS: EQ visual analogue scale; n: number of subjects;

SD: standard deviation; VAS: visual analog scale.

**Table S4. Treatment satisfaction assessed using TSQM-1.4 questionnaire by BPI-Interference score (<3 and ≥3)**

| **Characteristics** | **All Patients Mean (SD)** | **Pain Interference <3 (n=339) Mean (SD)** | **Pain Interference ≥3 (n=262) Mean (SD)** | **P value** |
| --- | --- | --- | --- | --- |
| TSQM-Effectiveness | 54.2 (14.1) | 57.45 (12.45) | 49.97 (15.04) | <0.0001 |
| TSQM-Side Effects | 96 (10.9) | 96.29 (10.60) | 95.63 (11.36) | 0.4673 |
| TSQM-Convenience | 62.3 (10.4) | 63.34 (10.65) | 60.96 (9.97) | 0.0059 |
| TSQM-Global Satisfaction | 59 (15.4) | 59.44 (13.44) | 58.34 (17.68) | 0.3907 |

BPI: Brief Pain Inventory; n: number of subjects; SD: standard deviation; TSQM: Treatment Satisfaction Questionnaire for

Medication (full score: 100).

**Table S5: The association between BPI-Interference score and HRQoL**

| **EQ-5D-5L and Self-assessed health per BPI-Interference score** | **Parameter estimate^a^** | **95% CI** | **P value** | **Parameter estimate^b^** | **95% CI** | **P value** |
| --- | --- | --- | --- | --- | --- | --- |
| EQ-5D-5L | -0.10 | (-0.1, -0.09) | 0.5458 | -0.09 | (-0.1, -0.09) | <0.0001 |
| Self-assessed health | -3.69 | (-4.35, -3.03) | <0.0001 | -3.38 | (-4.06, -2.7) | <0.0001 |

aAdjusted for age. bAdjusted for age, sex, BMI, number of pain sites, and comorbidity. BMI: body mass index;

BPI: Brief Pain Inventory; CI: confidence interval; EQ-5D-5L: EQ 5 dimension-5-level;

HRQoL: health-related quality of life.

**Table S6: The association between BPI-Interference score and TSQM**

| **TSQM per BPI-Interference score** | **Parameter estimate^a^** | **95% CI** | **P value** | **Parameter estimate^b^** | **95% CI** | **P value** |
| --- | --- | --- | --- | --- | --- | --- |
| TSQM-effectiveness | -3.12 | (-3.75, -2.49) | <0.0001 | -3.32 | (-3.96, -2.67) | <0.0001 |
| TSQM-side effect | -0.52 | (-1.03, 0) | 0.0513 | -0.56 | (-1.09, -0.02) | 0.0416 |
| TSQM-convenience | -0.94 | (-1.43, -0.44) | 0.0002 | -0.84 | (-1.35, -0.33) | 0.0014 |
| TSQM-global satisfaction | -0.52 | (-1.26, 0.22) | 0.1704 | -0.74 | (-1.49, 0.01) | 0.0543 |

aAdjusted for age. bAdjusted for age, sex, BMI, number of pain sites, and comorbidity. BMI: body mass index;

BPI: Brief Pain Inventory; CI: confidence interval; TSQM: Treatment Satisfaction Questionnaire for Medication.

**Table S7: The association between BPI-Interference score and HRQoL in patients with/without comorbidity**

| **EQ-5D-5L and Self-assessed health per BPI-Interference score** | **With comorbidity** | | | |  | **Without comorbidity** | | | | | **P value^c^** |
| --- | --- | --- | --- | --- | --- | --- | --- | --- | --- | --- | --- |
|  | **Parameter estimate^a^** | **95% CI** | **Parameter estimate^b^** | **95% CI** |  | **Parameter estimate^a^** | **95% CI** | **Parameter estimate^b^** | | **95% CI** |  |
| EQ-5D-5L | -0.09 | (-0.1, -0.09) | -0.09 | (-0.1, -0.08) |  | -0.10 | (-0.11, -0.09) | -0.10 | (-0.11, -0.09) | | 0.5523 |
| Self-assessed health | -3.52 | (-4.33, -2.72) | -3.19 | (-4.01, -2.37) |  | -4.03 | (-5.21, -2.85) | -3.82 | (-5.04, -2.6) | | 0.3780 |

aAdjusted for age. bAdjusted for age, sex, BMI, and number of pain sites. cAdjusted for age, sex, BMI, number of pain sites, and comorbidity. BMI: body mass index; BPI: Brief Pain Inventory; CI: confidence interval; EQ-5D-5L: EQ 5 dimension-5-level; HRQoL: health-related quality of life.

# Table S8. The association between BPI-Interference score and TSQM in patients with/without comorbidity

| **TSQM per BPI-Interference score** | **With comorbidity** | | | |  | **Without comorbidity** | | | | | | **P value^c^** |
| --- | --- | --- | --- | --- | --- | --- | --- | --- | --- | --- | --- | --- |
|  | **Parameter estimate^a^** | **95% CI** | **Parameter estimate^b^** | **95% CI** |  | **Parameter estimate^a^** | **95% CI** | | **Parameter estimate^b^** | | **95% CI** |  |
| TSQM-effectiveness | -2.84 | (-3.62, -2.06) | -2.99 | (-3.8, -2.18) |  | -3.64 | | (-4.74, -2.54) | | -3.95 | (-5.07, -2.83) | 0.2531 |
| TSQM-side effect | -0.27 | (-0.92, 0.37) | -0.37 | (-1.04, 0.29) |  | -0.94 | | (-1.82, -0.05) | | -0.88 | (-1.8, 0.04) | 0.2964 |
| TSQM-convenience | -1.02 | (-1.65, -0.39) | -0.95 | (-1.61, -0.3) |  | -0.79 | | (-1.6, 0.02) | | -0.64 | (-1.48, 0.19) | 0.7364 |
| TSQM-global satisfaction | -0.43 | (-1.33, 0.47) | -0.69 | (-1.61, 0.24) |  | -0.40 | | (-1.69, 0.9) | | -0.72 | (-2.04, 0.6) | 0.9169 |

aAdjusted for age. bAdjusted for age, sex, BMI, and number of pain sites. cAdjusted for age, sex, BMI, number of pain sites, and comorbidity. BMI: body mass index; BPI: Brief Pain Inventory; CI: confidence interval; TSQM: Treatment Satisfaction Questionnaire for Medication.
